# Propofol postconditioning ameliorates hypoxia/reoxygenation induced H9c2 cell apoptosis and autophagy via upregulating forkhead transcription factors under hyperglycemia

**DOI:** 10.1186/s40779-021-00353-0

**Published:** 2021-11-10

**Authors:** Rong-Hui Han, He-Meng Huang, Hong Han, Hao Chen, Fei Zeng, Xiang Xie, Dan-Yong Liu, Yin Cai, Liang-Qing Zhang, Xin Liu, Zheng-Yuan Xia, Jing Tang

**Affiliations:** 1 Department of Anesthesiology, Affiliated Hospital of Guangdong Medical University, Guangdong, 57 South Renming Avenue Xiashan District, Zhanjiang City, 524000 Guandong Province China; 2 Department of Emergency, Affiliated Hospital of Guangdong Medical University, Zhanjiang, 524000 China; 3 Department of Anesthesiology, the Eighth Affiliated Hospital, Sun Yat-Sen University, Guangzhou, 518000 China; 4 Department of Anesthesiology, Guangzhou First People’s Hospital, The Second Affiliated Hospital of South China University of Technology, Guangzhou, 510000 China; 5 Department of Anesthesiology, The Second Affiliated Hospital and Yuying Children’s Hospital, Wenzhou Medical University, Wenzhou, 325000 China; 6 Department of Health Technology and Informatics, The Hong Kong Polytechnic University, Hung Hom, 999077 Hong Kong SAR China; 7 State Key Laboratory of Pharmaceutical Biotechnology, Department of Medicine, The University of Hong Kong, Pok Fu Lam, 999077 Hong Kong SAR China

**Keywords:** H/R injury, Hyperglycemia, P-PostC, Apoptosis, Autophagy, FoxO

## Abstract

**Background:**

Administration of propofol, an intravenous anesthetic with antioxidant property, immediately at the onset of post-ischemic reperfusion (propofol postconditioning, P-PostC) has been shown to confer cardioprotection against ischemia–reperfusion injury, while the underlying mechanism remains incompletely understood. The FoxO transcription factors are reported to play critical roles in activating cardiomyocyte survival signaling throughout the process of cellular injuries induced by oxidative stress and are also involved in hypoxic postconditioning mediated neuroprotection, however, the role of FoxO in postconditioning mediated protection in the heart and in particular in high glucose condition is unknown.

**Methods:**

Rat heart-derived H9c2 cells were exposed to high glucose (HG) for 48 h (h), then subjected to hypoxia/reoxygenation (H/R, composed of 8 h of hypoxia followed by 12 h of reoxygenation) in the absence or presence of postconditioning with various concentrations of propofol (P-PostC) at the onset of reoxygenation. After having identified the optical concentration of propofol, H9c2 cells were subjected to H/R and P-PostC in the absence or presence of FoxO1 or FoxO3a gene silencing to explore their roles in P-PostC mediated protection against apoptotic and autophagic cell deaths under hyperglycemia.

**Results:**

The results showed that HG with or without H/R decreased cell viability, increased lactate dehydrogenase (LDH) leakage and the production of reactive oxygen species (ROS) in H9c2 cells, all of which were significantly reversed by propofol (P-PostC), especially at the concentration of 25 µmol/L (P25) (all *P* < 0.05, NC *vs*. HG; HG *vs.* HG + HR; HG + HR + P12.5 or HG + HR + P25 or HG + HR + P50 *vs.* HG + HR). Moreover, we found that propofol (P25) decreased H9c2 cells apoptosis and autophagy that were concomitant with increased FoxO1 and FoxO3a expression (all *P* < 0.05, HG + HR + P25 *vs*. HG + HR). The protective effects of propofol (P25) against H/R injury were reversed by silencing FoxO1 or FoxO3a (all *P* < 0.05, HG + HR + P25 *vs.* HG + HR + P25 + siRNA-1 or HG + HR + P25 + siRNA-5).

**Conclusion:**

It is concluded that propofol postconditioning attenuated H9c2 cardiac cells apoptosis and autophagy induced by H/R injury through upregulating FoxO1 and FoxO3a under hyperglycemia.

## Background

Numerous studies have demonstrated that the reestablishment of coronary blood flow to ischemic heart diseases is the most effective treatment to rescue the damage of ischemic myocardium. It is worth pointing out, however, that reperfusion of the ischemic myocardium could result in secondary damage, which is termed ischemic reperfusion (I/R) injury [1–3]. Diabetes mellitus (DM), has always been the focus of attention stem from various complications which could erode organs (especially heart and kidney) and tissues through disarranging the intracellular signaling as well as increasing the susceptibility to cell injuries. Diabetes and/or hyperglycemia will increase the risk of ischemic heart disease and the severity of ischemic heart disease and I/R injury that may be associated with or proportional to the levels of blood sugar [4–7].

Oxidative stress, an imbalanced condition, irritated by massive burst production of reactive oxygen species (ROS) in the absence of sufficient antioxidant reservation during myocardial reperfusion has been substantiated to play an imperative role in causing and aggravating I/R injury. Oxidative stress also acts as a signal to induce apoptosis, autophagy and necrosis during acute ischemia–reperfusion injury [8–12]. Among all these manners of cell death, apoptosis appears to be an inseparable part in the elimination of invalid cells and it's evidenced that the reduction in the expression of apoptosis-inducing Bax gene is beneficial to myocardial I/R injury [13]. However, it is debatable regarding whether or not autophagy is a beneficial effector mechanism of cell death, since the study has shown an advantageous effect on the pathological process of I/R injury by inhibiting autophagy via regulating Beclin1, while the disadvantageous effect was also observed via fading the expression of Atg5 gene [14, 15].

How to effectively maintain the intracellular ROS homeostasis and to restore the antioxidant balance through intrinsic and extrinsic pathways has become a focus in many fields [16, 17]. Due to the speedy onset, rapid and complete recovery characteristics, propofol (2–6-diisopropylphenol) is widely utilized in various surgical procedures and intensive care units. Animal models and clinical studies have shown that apart from its anesthetic effects, propofol exerts protective effects against myocardial I/R injury [18, 19]. The mechanisms involved the elimination of ROS, inhibition of apoptosis, maintenance of mitochondrial function, reduction of inflammatory mediators, inhibition of excessive autophagy or induction of moderate autophagy [20–24]. However, the potential molecular mechanism by which propofol may exert cardioprotective effects on diabetic myocardial I/R injury has not been fully elucidated, and propofol has been shown to have cytotoxic effects in some cases [25, 26] especially when used as relatively high concentrations [27].

In recent years, studies have demonstrated that members of the class O of forkhead box transcription factors (FoxO) play critical roles in cellular homeostasis, including metabolism, cellular proliferation, oxidative stress response, and cell death. FoxOs consist of four members, FoxO1, FoxO3a, FoxO4 and FoxO6. So far, two subtypes of FoxO (FoxO1 and FoxO3a) are known to be essential for the maintenance of cardiovascular homeostasis [28–30]. Pathological conditions such as inflammation, I/R and metabolic abnormalities are often accompanied by different expressions of FoxO1 [31, 32]. It has been shown that propofol increases FoxO1 expression in a dose-dependent manner to exert its cardiac protective effects that involved the attenuation of oxidative stress [33]. In contrast, in the heart of diabetic rats, deletion of FoxO1 increased caspase-dependent apoptosis [34]. FoxO3a is the intersection of multiple signaling pathways in oxidative stress, and FoxO3a is highly expressed in the heart. In vivo experiments show that FoxO3a can reduce oxidative stress by increasing the expression of manganese superoxide dismutase (MnSOD) and the production of catalase and peroxidase III [35]. When FoxO3a was knocked out, even the overexpression of SIRT1, a protein that stimulates antioxidant genes, could not increase the expression of antioxidant enzymes. However, a study has also shown that ROS activates FoxO3a by inhibiting Akt and accelerates cardiac microvascular endothelial death [36]. All these collectively suggest that FoxO1 and FoxO3a play important roles in a wide variety of cellular processes, and the exact roles they play may vary depending on disease conditions.

Specific questions remain regarding whether FoxO1 and FoxO3a may mediate the cardioprotective effect of propofol postconditioning (P-PostC) on myocardial I/R injury under hyperglycemia, or whether P-PostC can reduce cellular oxidative stress by regulating the expression of FoxO1 and/or FoxO3a to protect the diabetic heart remains unclear. In the current study, we speculated that P-PostC may attenuate hypoxia/ reoxygenation-induced apoptosis and autophagy by regulating the expression of FoxO1 and FoxO3a in H9c2 cell under hyperglycemia.

## Methods

### Reagents and antibodies

DMSO (dimethyl sulfoxide) and Propofol (2, 6-Diisopropylphenol, 97%) were purchased from Sigma Aldrich. All SDS-PAGE reagents, ECL Western Blotting Substrate and Paraformaldehyde (4%) reagents were from Solarbio company (China). All culture reagent and transfection reagent were from Thermo-Invitrogen (Massachusetts, America). The FoxO1 and FoxO3a siRNAs were designed and provided by Guangzhou Ribibio (China). Experimental kits were as follows: cell counting kit-8 (CCK-8) (Japan Tongren), lactate dehydrogenase (LDH) release assay kit (Roche), malondialdehyde (MDA) ELISA kit (rat), total superoxide dismutase (T-SOD) ELISA kit (rat), creatine kinase-MB (CK-MB) ELISA kit (rat) and cardiac troponin I (cTnI) ELISA kit (rat, all from Jonln company, China), Dihydroethidium (DHE) assay Kit, Bisbenzimide Hoechst (Hoechst 33,258), ROS assay kit and Annexin V-FITC apoptosis assay kit (all from Beyotime company, China). Rabbit antibodies against FoxO1, FoxO3a, β-actin, Bax, caspase-3, cleaved caspase-3, p62, Beclin1 and HRP-conjugated secondary antibodies were from Cell Signaling Technology (CST) while antibodies for Bcl-2, LC3B were purchased from Sigma Aldrich.

### Cell culture and treatment protocol

The H9c2 cells, a cardiac cell strain derived from the S-D Rat left ventricle, were purchased from the Shanghai Institute for Biological Sciences, Chinese Academy of Sciences (Shanghai, China). The cells (2 × 10^5^ cells per well in a six-well culture plate) were cultured in Dulbecco's modified eagle medium (DMEM) supplemented with 10% fetal bovine serum, 100 U/ml penicillin and 100 μg/ml streptomycin. Cells were grown in a humidified incubator consisting of 95% air and 5% CO_2_ at 37 °C.

Hypoxia/Reoxygenation (H/R) model and drug treatment were performed similarly as previously described [37]. In brief, cells were exposed to hyperglycemia (35 mM glucose) for 48 h before being subjected to hypoxia conditions (oxygen deprivation) in glucose and fetal bovine serum free cultivation medium. After 8 h of oxygen deficit, H9c2 cells were reoxygenated for 12 h in hyperglycemia medium. Different concentrations of Propofol (graded from low to high concentration varying from 12.5, 25, 50 to 100 μmol/L) and DMSO (100 μmol/L) were applied respectively to the cells during the reoxygenation.

### FoxO1/FoxO3a-Specific siRNA transfection

H9c2 Cells were plated at the density of 5 × 10^4^ cells/well in each 6-well plate and incubated in eligible cultivation cabinet. Based on the manufacturer’s manual, specifically designed and synthetic siRNA (50 nmol/L) of FoxO1 or FoxO3a (provided by the Guangzhou Ribibio company) were separately transfected into cells with the recommended agent (Lipofectamine 2000) in the next morning to silence FoxO1 or FoxO3a expression. Six hours after transfection, H9c2 cells were refreshed with hyperglycemia cell culture medium for 48 h and were then assigned to continue the experiments. The FoxO1and FoxO3a specific siRNA: FoxO1-si-1: 5′- GAATTCAATTCGCCACAAT -3′; FoxO1-si-2: 5′-CATGGACAACAACAGTAAA-3′; FoxO1-si-3: 5′-GGAGAACCTTCTGGATAAT-3′; FoxO3a-si-1: 5′-GCTCTTGGTGGATCATCAA-3′; FoxO3a-si-2: 5′-CCTCATGGATGACCTGCTA-3′; FoxO3a-si-3: 5′-GGAACGTGATGCTTCGCAA-3′.

### Cell viability assay

CCK8, a fast and highly sensitive testing kit based on WST-8 that could turn into orange (the color is linearly related to the number and viability of cells) formazan by dehydrogenase of mitochondria. At the same time, LDH Release Assay Kit was used to detect the degree of cytotoxicity as the reflection of cell membrane damage. H9c2 cells were seeded into 96-well culture plates (8000 cells/well), after precedent experiment conditioning, all cells and cells culture supernatant were processed according to the procedures of the relevant manufactures. Then we used a microplate reader to measure the OD (optical density) value at a suitable wavelength (450 nm) which indirectly reflects the magnitude of viabilities and damage of cells.

### Detection of cTnI, CK-MB, MDA and T-SOD

Cells were seeded into a 6-well plate at a density of 1 × 10^5^ cells/well. After 48 h of hyperglycemic cultivation, H9c2 cells were subjected to hypoxia for 8 h and subsequently disposed of different concentrations of propofol during reoxygenation condition for 12 h. To evaluate the degree of myocardial cellular injury (release of CK-MB and cTnI) and intracellular ROS (release of MDA and T-SOD), a double antibody sandwich Enzyme-linked immunosorbent assay was applied for these tests. The cells supernatant in all groups and related reagents were added to above ELISA kits sequently by protocols. A microplate reader was used to measure the absorbance at the 450 nm wavelength to estimate the degree of cell injury and ROS.

### Measurement of reactive oxygen species

Cells were cultured into a 6-well plate and treated as noted mentioned above. DHE and ROS assay kit were used for the detection of oxidative stress-induce cellular damages according to the manufacturer’s specifications. DHE is one of the most commonly used fluorescent probes of ROS, as ethidium can bind to RNA or DNA tightly to produce red fluorescence depending on the amounts of superoxide anion. DCFH-DA (2, 7-dichlorodihydrofluorescein diacetate) ROS Assay Kit also is one of the most sensitive fluorescent probes. The results of DCFH-DA were measured and analyzed by a flow cytometer.

### Assessment of apoptosis

H9c2 cells were plated into normal culture plates and manipulated as mentioned before. Annexin V-FITC apoptosis detection kit and Hoechst 33,258 were used for the detection of apoptotic cells according to the manufacturer’s description. The apoptotic index was calculated as the ratio of the number of apoptotic cells to total cells.

### Western blotting

After the completion of treatments, cells from each group were collected and washed twice and then harvested gently with precooled phosphate buffered saline (PBS) which then was removed by centrifugation at 3000 rpm for 10 min at 4 °C. The sedimentation of cells was lysed with Radio Immunoprecipitation Assay (RIPA) buffer containing protease and phosphatase inhibitor cocktails in the ice bath for 20 min. Then soluble protein samples were collected by centrifugation at 10,000 rpm for 10 min at 4 °C and quantified for equal concentration with PBS and loading buffer by the protocol of Bicinchoninic Acid (BCA) kit. The same volume of protein from each group was added and isolated by 10–12% SDS polyacrylamide gel and then transferred to a PVDF membrane. After 2 h of blocking with 5% no-fat milk, the PVDF membrane was separated and incubated with matching antibodies (including FoxO1, FoxO3a, β-actin, Bcl-2, Bax, caspase-3, cleaved caspase-3, p62, Beclin1, LC3B) on a shaking platform for 12–16 h at 4 °C. After primary antibody incubation, the membranes were washed with TBST and incubated with HRP-conjugated secondary antibodies for 1–2 h at room temperature. The PVDF membranes were exposed with ECL reagent in a photographic processor after washing. Data are presented as percent change relative to β-actin used as a reference.

### Statistical analysis

All the values are presented as means ± standard error of mean (SEM). And the comparison between groups was made with one-way analysis of variance (ANOVA) followed by Turkey's test while multiple comparisons were analyzed by Bonferroni's correction. *P* < 0.05 was considered statistically significant.

## Results

### Propofol-postconditioning (P-PostC) dose dependently alleviated hypoxia/reoxygenation injury in H9c2 cells under hyperglycemia

**Fig. 1:**
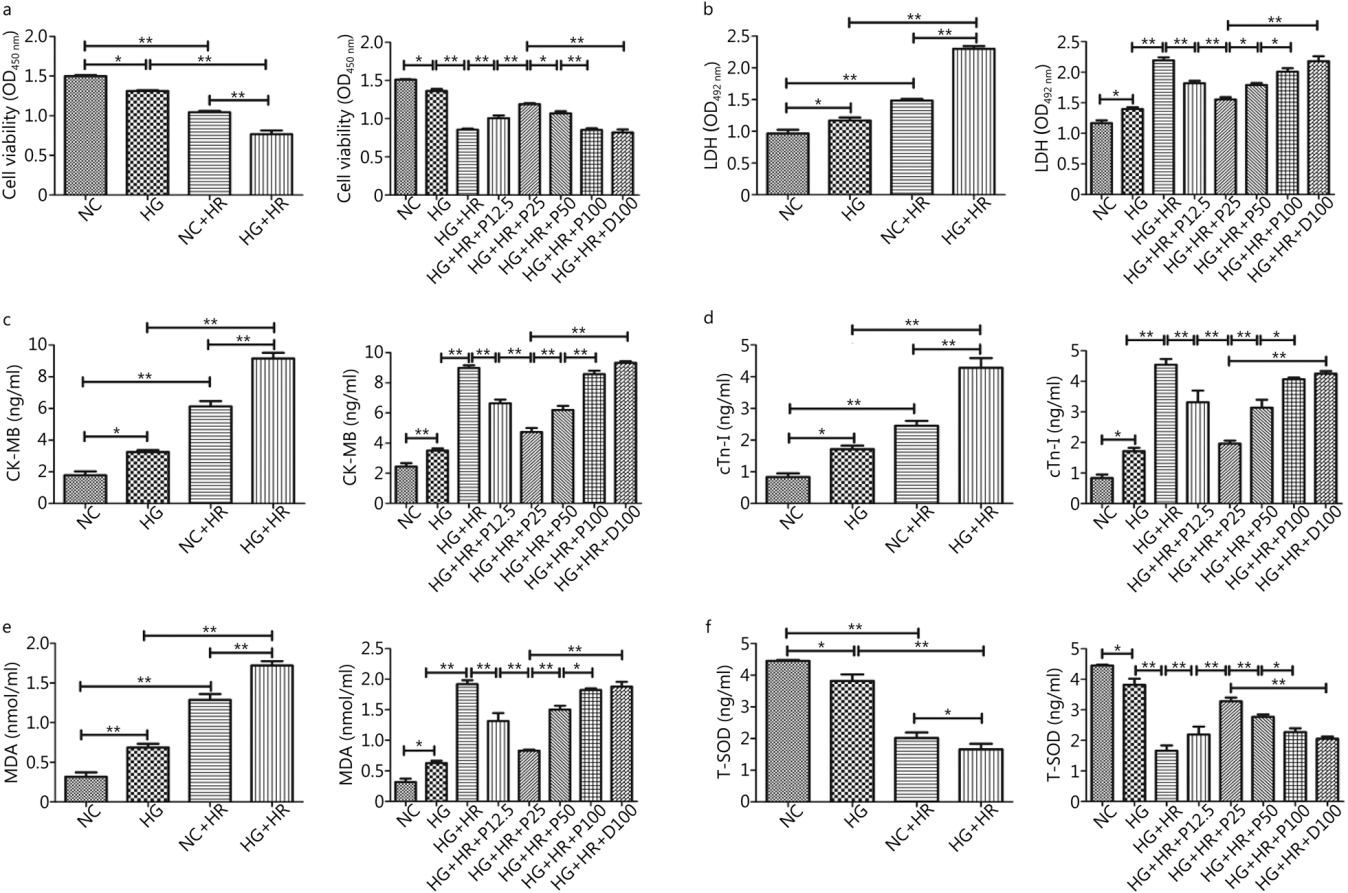
Effects of P-PostC (propofol-Postconditioning) on HR (hypoxia/reoxygenation) induced H9c2 cell injury under hyperglycemia (35 mmol/L). **a** Cell viability. **b** Leakage of LDH (lactate dehydrogenase). **c** Concentrations of CK-MB (creatine kinase-MB). **d** Extent of cTnI (cardiac troponin I). **e** Degree of MDA (malondialdehyde). **f** Level of T-SOD (total superoxide dismutase). Data are expressed as mean ± SEM from three independent experiments each were performed in triplicate. ^*^P < 0.05, ^**^P < 0.01

We used the in vitro Hypoxia/Reoxygenation (H/R) model in H9c2 cells exposed to high glucose to simulate the process of ischemia/reperfusion in diabetes. As shown in Fig. 1, cell viability (Fig. 1a) and T-SOD (Fig. 1f) were significantly reduced when cells were exposed to hyperglycemia (HG group) alone as compared to the normal glucose group (NC group). And the reductions in cell viability and T-SOD were further exaggerated by H/R condition (HG + HR group), along with the augmentations of LDH (Fig. 1b), CK-MB (Fig. 1c) and cTnI release (Fig. 1d) and elevated MDA production (Fig. 1e) and reduction in T-SOD. Meanwhile, we found that P-PostC attenuated above cell damages in a dose-dependent manner, being effective at concentrations ranging from 12.5 to 50 µmol/L while the most prominent effect was seen at 25 µmol/L (all *P* < 0.05, NC *vs*. HG; HG *vs.* HG + HR; HG + HR + P12.5 or HG + HR + P25 or HG + HR + P50 *vs.* HG + HR, Fig. 1a to 1f). Besides, no apparent protective effects were seen in propofol concentration at 100 µmol/L (HG + HR + P100) group or in DMSO (HG + HR + D100) compared to HG + HR group (all* P* > 0.05, HG + HR + P100 or HG + HR + D100 *vs.* HG + HR).

### P-PostC attenuated the H/R induced increases in ROS production and apoptotic cell death under high glucose

**Fig. 2:**
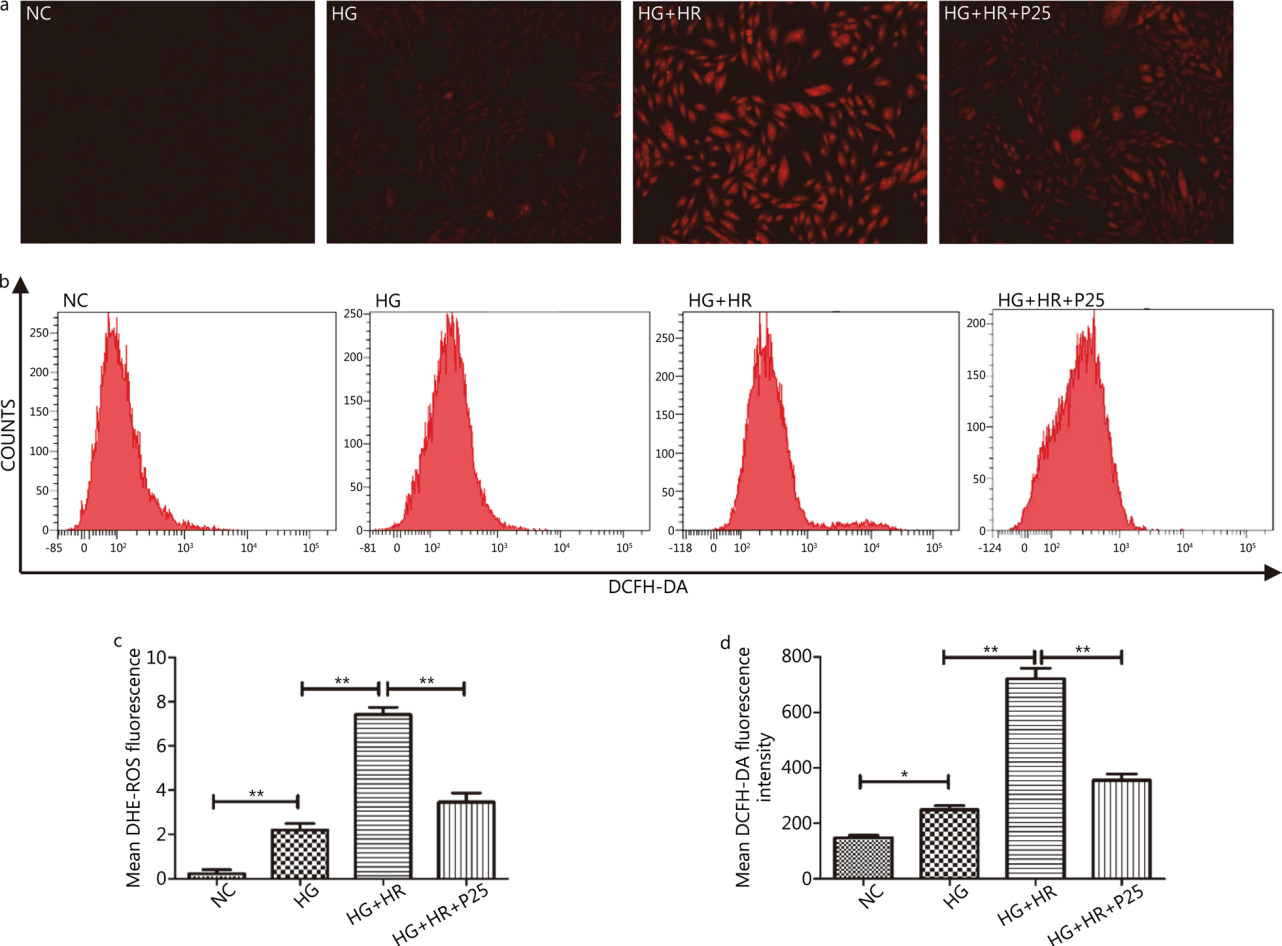
Effects of P-PostC (propofol-Postconditioning) on ROS (reactive oxygen species) Levels during HR (hypoxia/reoxygenation) injury under hyperglycemia (35 mmol/L). **a** ROS degrees measured by DHE (dihydroethidium) (100 ×). **b** ROS levels detected by DCFH-DA (2, 7-dichlorodihydrofluorescein diacetate) fluorescence flow cytometry. **c** Statistical graph of DHE. **d** Statistical image of mean DCFH-DA fluorescence intensity. Data are shown as mean ± SEM from three independent experiments each performed in triplicate. ^*^P < 0.05, ^**^P < 0.01

**Fig. 3:**
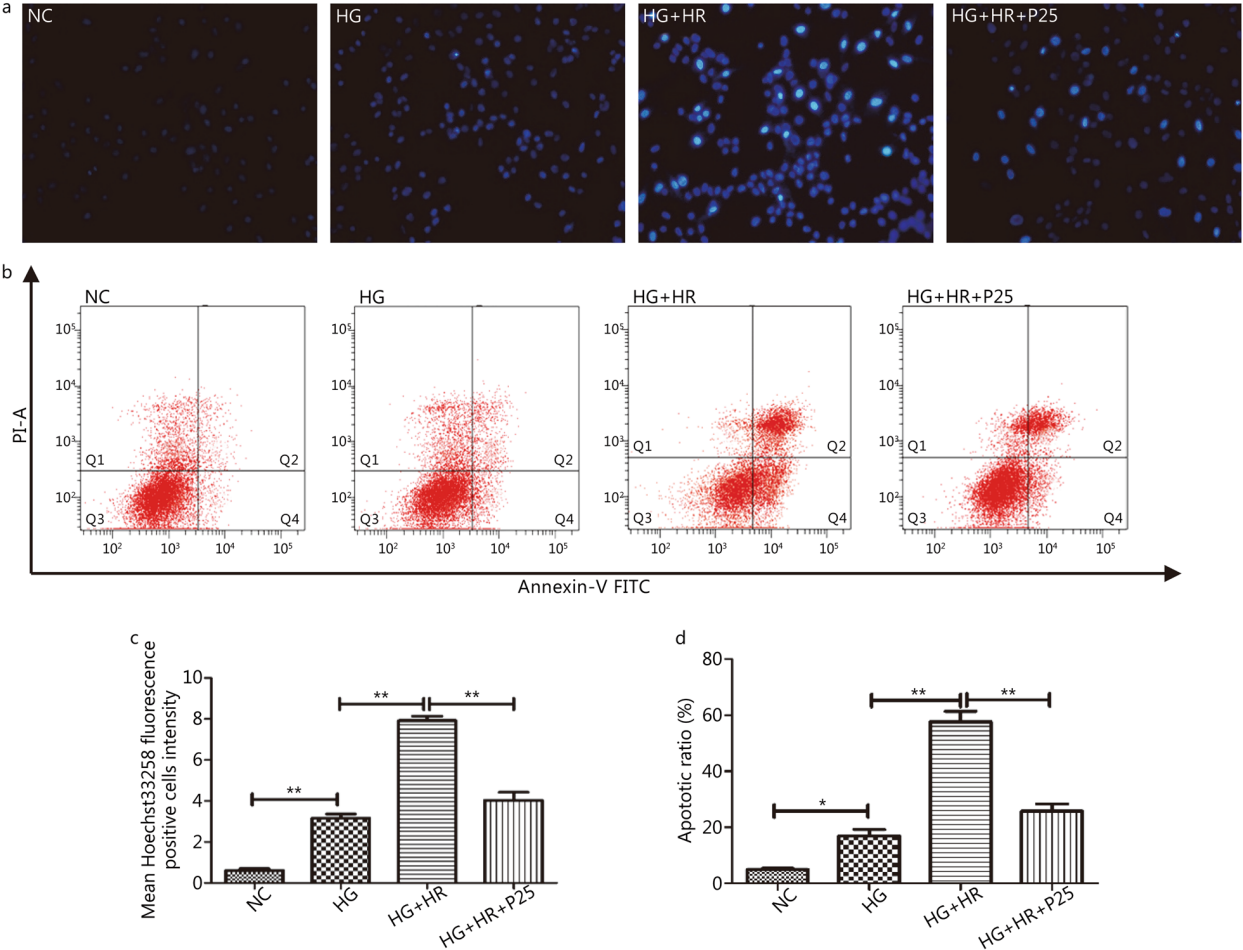
Inhibition of apoptotic ratio by P-PostC (propofol-Postconditioning) at the concentration 25 µmol/L in H9c2 cells subjected to H/R under hyperglycemia. **a** Apoptotic cells marked by Hoechst 33,258 staining (200 ×). **b** Counts of apoptotic cells by flow cytometry. **c** Mean Hoechst 33,258 fluorescence positive intensity. **d** Quantification of apoptotic ratio. Data are mean ± SEM from three independent experiments each performed in triplicate. ^*^P < 0.05, ^**^P < 0.01

To further investigate the mechanism whereby P-PostC ameliorated H/R injury under HG, we then examined the ROS levels and apoptosis using a fluorescent probe when cells were treated with propofol at 25 µmol/L. As depicted in Fig. 2, mean DHE-ROS measured by fluorescent microscope and DCFH-DA fluorescence intensity detected by flow cytometry were gradually increased from NC group to HG + HR group (all *P* < 0.05, NC *vs.* HG; HG *vs.* HG + HR), which were both significantly attenuated by P-PostC. Similarly, as shown in Fig. 3, P-PostC significantly attenuated the ratios of apoptotic cell death after H/R in H9c2 exposed to high glucose (all *P* < 0.05, NC *vs.* HG; HG *vs.* HG + HR; HG + HR *vs.* HG + HR + P25).

### P-PostC reduced apoptosis and autophagy in H9c2 cells subjected to H/R injury under high glucose

**Fig. 4:**
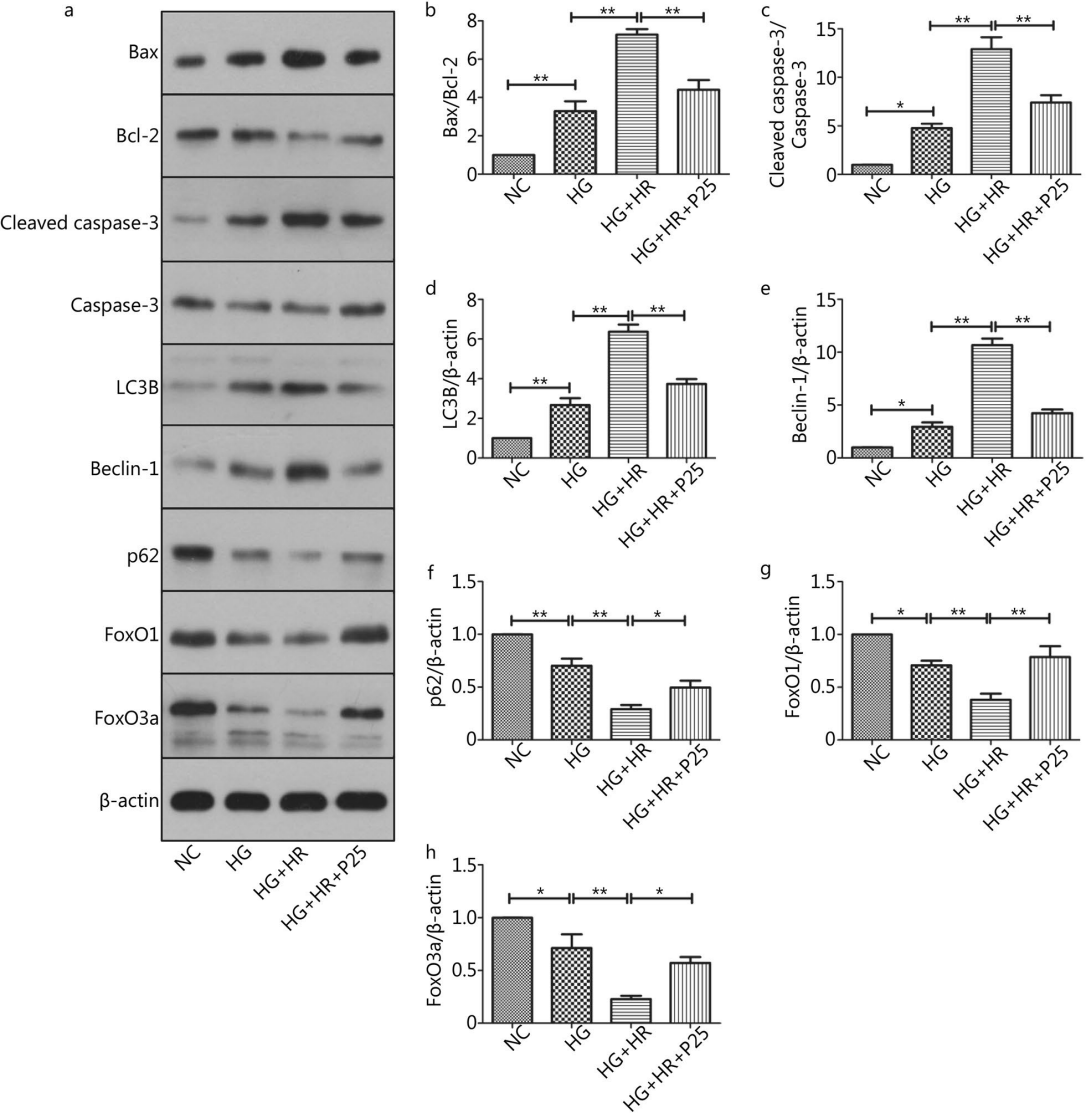
Anti-apoptotic and autophagy effects of P-PostC (propofol-Postconditioning) on HR (hypoxia/reoxygenation) injury under high glucose. **a** Representative images of protein expression. **b** Ratio of Bax/Bcl-2. **c** Ratio of cleaved caspase-3 to caspase-3. **d** Grey value of LC3B/β-actin. **e** Beclin-1 protein expression. **f** p62 protein expression. **g** FoxO1 protein expression. **h** FoxO3a protein expression. Data are mean ± SEM from three independent experiments each performed in triplicate. ^*^P < 0.05, ^**^P < 0.01

Since I/R injury involved various types of cell death pathways, so we measured the expression of apoptosis-associated and autophagy-related proteins using western blotting assay. As depicted in Fig. 4, high glucose alone or in the presence of H/R significantly enhanced the expression of apoptosis-promoting genes and inhibited the expression of apoptosis-restraining genes, including Bax/Bcl-2 and cleaved caspase-3/caspase-3. As such, the Bax/Bcl-2 ratio (Fig. 4a, b) and the ratio of and cleaved caspase-3/caspase-3 (Fig. 4a, c) in the HG + HR group was significantly higher than HG and HG + HR + P25 group (all *P* < 0.05, HG + HR *vs.* HG; HG + HR *vs.* HG + HR + P25). At the same time, the expression of autophagy-related genes protein, such as LC3B (Fig. 4d) and Beclin-1 (Fig. 4e) were activated, while the level of p62 was reduced (Fig. 4f). As such, the LC3B and Belcin-1 ratio in the HG + HR group was significantly higher than HG and HG + HR + P25 group (all *P* < 0.05, HG + HR *vs.* HG; HG + HR *vs.* HG + HR + P25) (Fig. 4a, d, e), and p62 in the HG + HR group was also significantly lower than HG and HG + HR + P25 group (all *P* < 0.05, HG + HR *vs.* HG; HG + HR *vs.* HG + HR + P25) (Fig. 4a, f). However, all these changes were reversed or significantly attenuated by P-PostC at the concentration of 25 µmol/L.

Previous studies suggested that FoxO family is essential for the maintenance of cardiovascular homeostasis and plays an important role in the regulation of cell death or survival signaling pathways. Thus, we examined the effect of propofol on FoxO family members, namely FoxO1 and FoxO3a, which are abundant in heart cells. As shown in Fig. 4g and h, the protein expression of both FoxO1 and FoxO3a were significantly reduced in H9c2 cells exposed to hyperglycemia (all *P* < 0.05, HG *vs*. NC) and were further significantly reduced when the cells were subjected to H/R (all *P* < 0.05, HG + HR *vs*. HG), and administration of propofol reverted H/R induced reductions in FoxO1 and FoxO3a expression (all *P* < 0.05, HG + HR + P25 *vs*. HG + HR).

### Silencing of FoxO1 or FoxO3a counteracted the protective effects of P-PostC against H/R injury of H9c2 cells exposed to high glucose

**Fig. 5:**
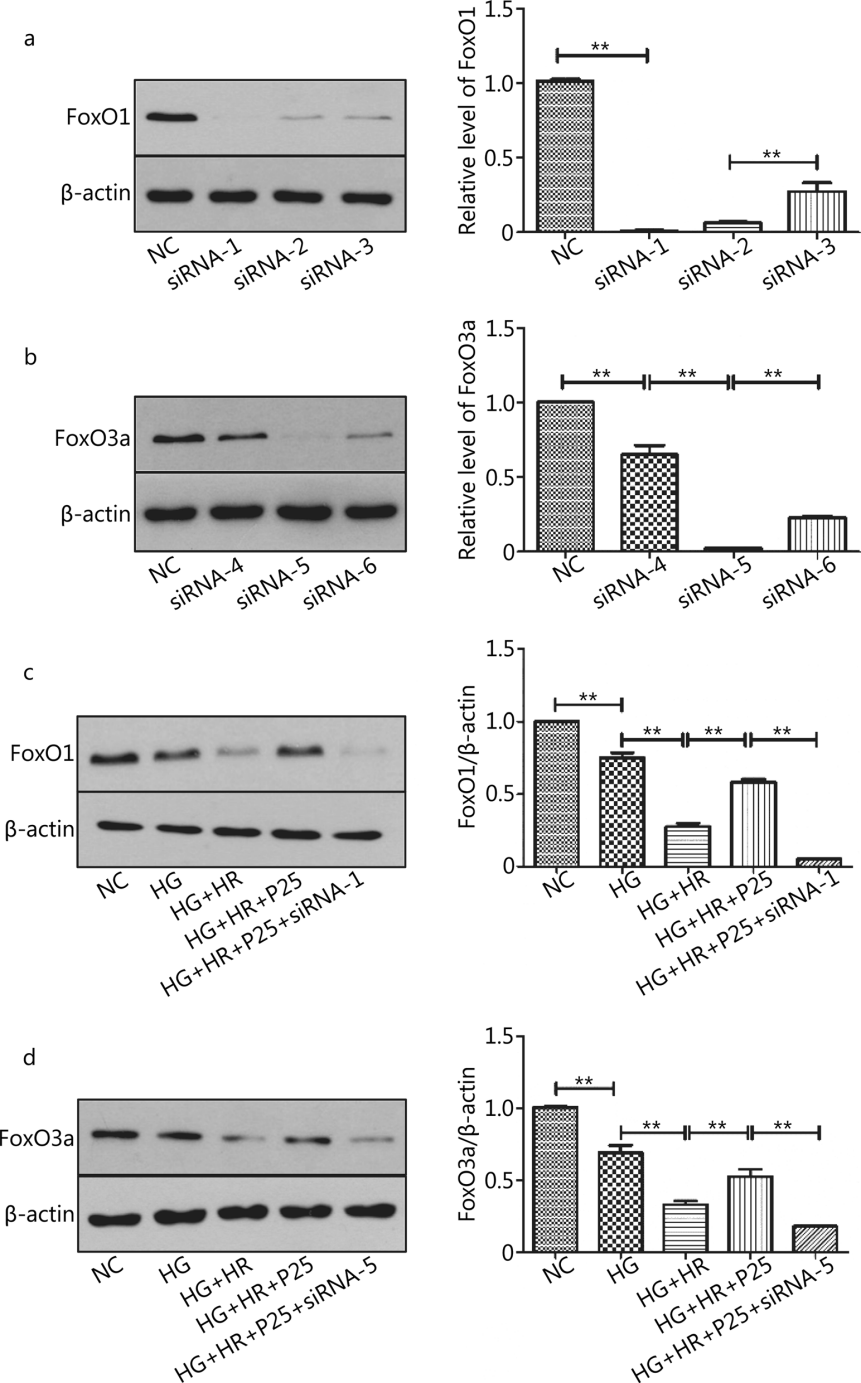
Effects of Silencing of FoxO1 or FoxO3a in the absence or presence of propofol in H9c2 cells subjected to HR (hypoxia/reoxygenation) under hyperglycemia. **a** Representative image of FoxO1 silence *vs* normal group. **b** Representative images of FoxO3a silence *vs* normal group. **c** Representative images of FoxO1 silence in HR model. **d** Representative images of FoxO3a silence in HR model. Data are mean ± SEM from three independent experiments each performed in triplicate. ^*^P < 0.05, ^**^P < 0.01

**Fig. 6:**
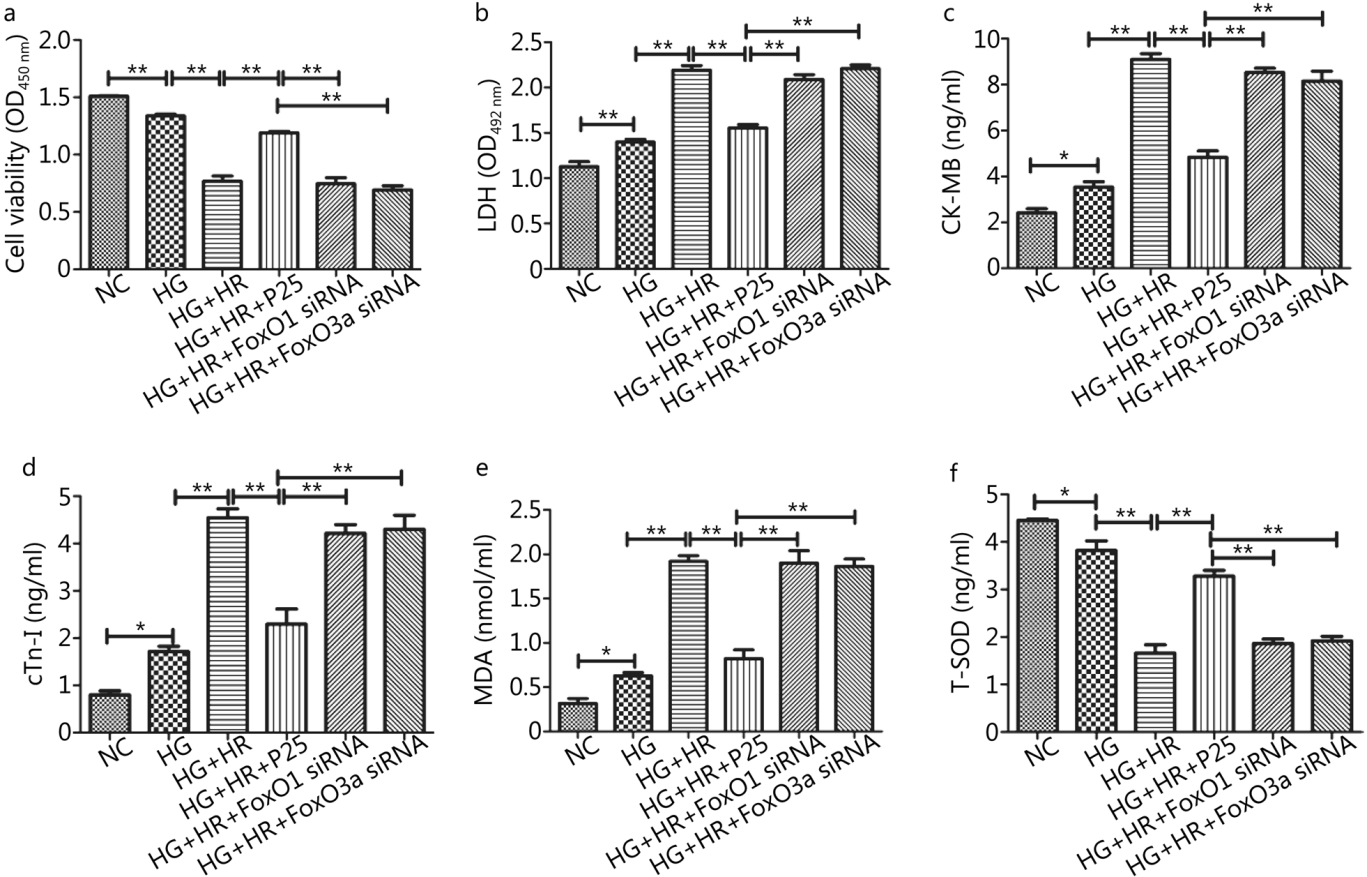
Disruption of FoxO1 or FoxO3a neutralized P-PostC (propofol Postconditioning) potency against cells injury subjected to HR (hypoxia/reoxygenation) under HG. **a** Cell viability. **b** LDH (lactate dehydrogenase) measured from supernatant of cells cultural medium. **c** Content of CK-MB (creatine kinase-MB). **d** Content of cTnI (cardiac troponin I). **e** Content of MDA (malondialdehyde). **f** Content of T-SOD (total superoxide dismutase). Data are mean ± SEM from three independent experiments each performed in triplicate. ^*^P < 0.05, ^**^P < 0.01

To explore the role of FoxO1 and FoxO3a in propofol mediated cellular protection against H/R injury, we specifically designed and synthesized three sequences of siRNAs respectively for FoxO1 and FoxO3a, namely siRNA-FoxO1 (siRNA 1–3) or siRNA-FoxO3a (siRNA 4–6). It turned out that transfection with siRNA-FoxO1-1 most profoundly downregulated the protein expression level of FoxO1 (Fig. 5a), while siRNA-FoxO3a–5 most profoundly downregulated FoxO3a (Fig. 5b). Then we knocked down FoxO1 and FoxO3a respectively in H9c2 cells respectively with siRNA-FoxO1-1 or siRNA-FoxO3a-5 (Fig. 5c, d) and assessed the efficacy of propofol on H/R injury under HG in the presence of FoxO1 or FoxO3a knockdown (Fig. 6). As shown in Fig. 5 c-d and Fig. 6, cell injury was further exacerbated when FoxO1 or FoxO3a was knocked down despite the presence of propofol treatment (all *P* < 0.05, HG + HR + P25 + siRNA1 *vs*. HG + HR + P25 or HG + HR; HG + HR + P25 + siRNA5 *vs*. HG + HR + P25 or HG + HR).

### FoxO1 or FoxO3a silencing canceled the protective effects of P-PostC against H/R injury in H9c2 cells exposed to high glucose

**Fig. 7:**
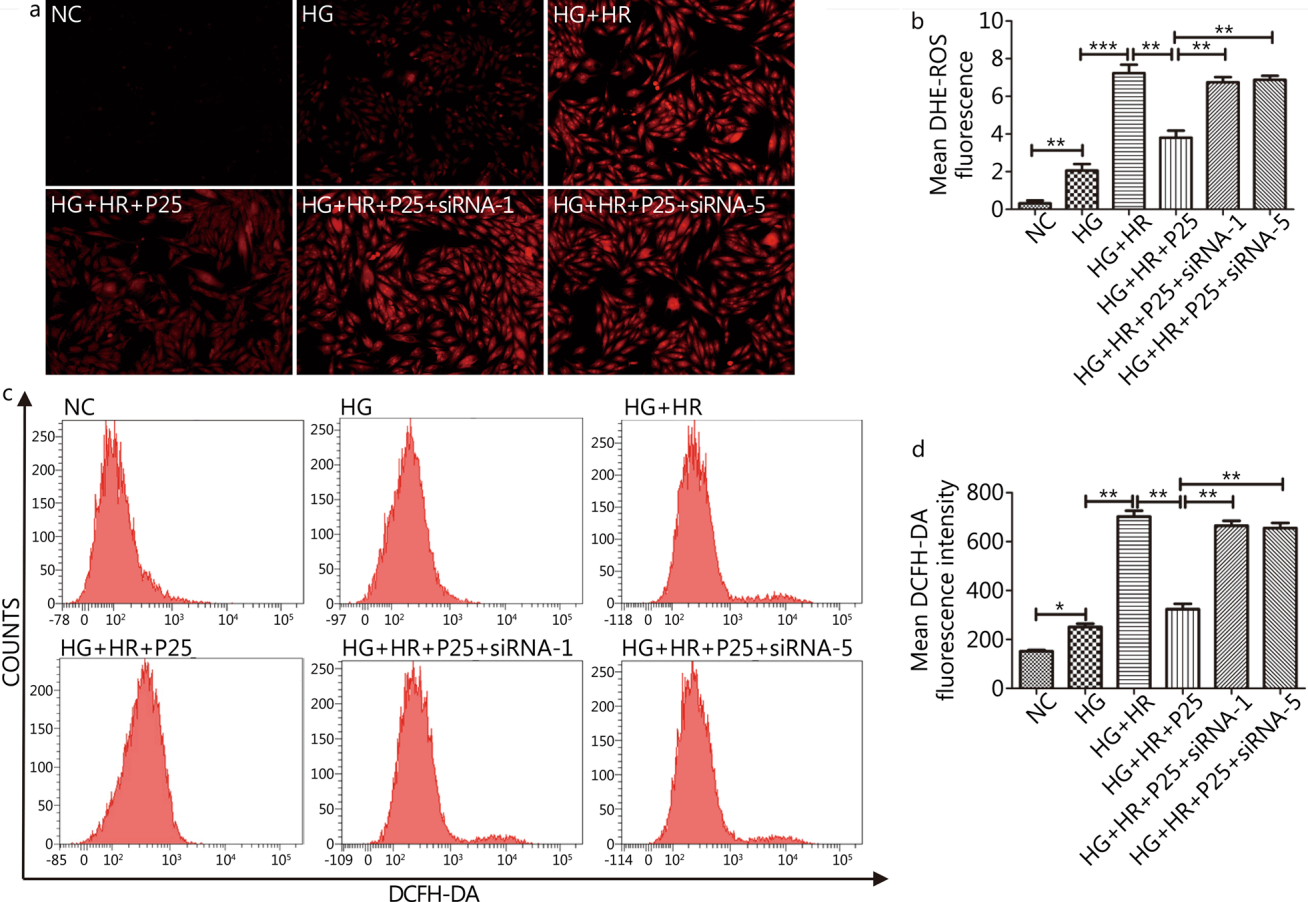
Knockout of propofol FoxO1 or FoxO3a canceled the protective effects of propofol in attenuating the augmentation of ROS Levels caused by H/R injury under hyperglycemia (35 mmol/L). **a** ROS (reactive oxygen species) degrees measured by DHE (dihydroethidium) fluorescence staining (100 ×). **b** ROS levels detected by DCFH-DA (2, 7-dichlorodihydrofluorescein diacetate) fluorescence flow cytometry. **c** Statistical graph of DHE. **d** Statistical image of mean DCFH-DA fluorescence intensity. Data are mean ± SEM from three independent experiments each performed in triplicate. ^*^P < 0.05, ^**^P < 0.01

**Fig. 8:**
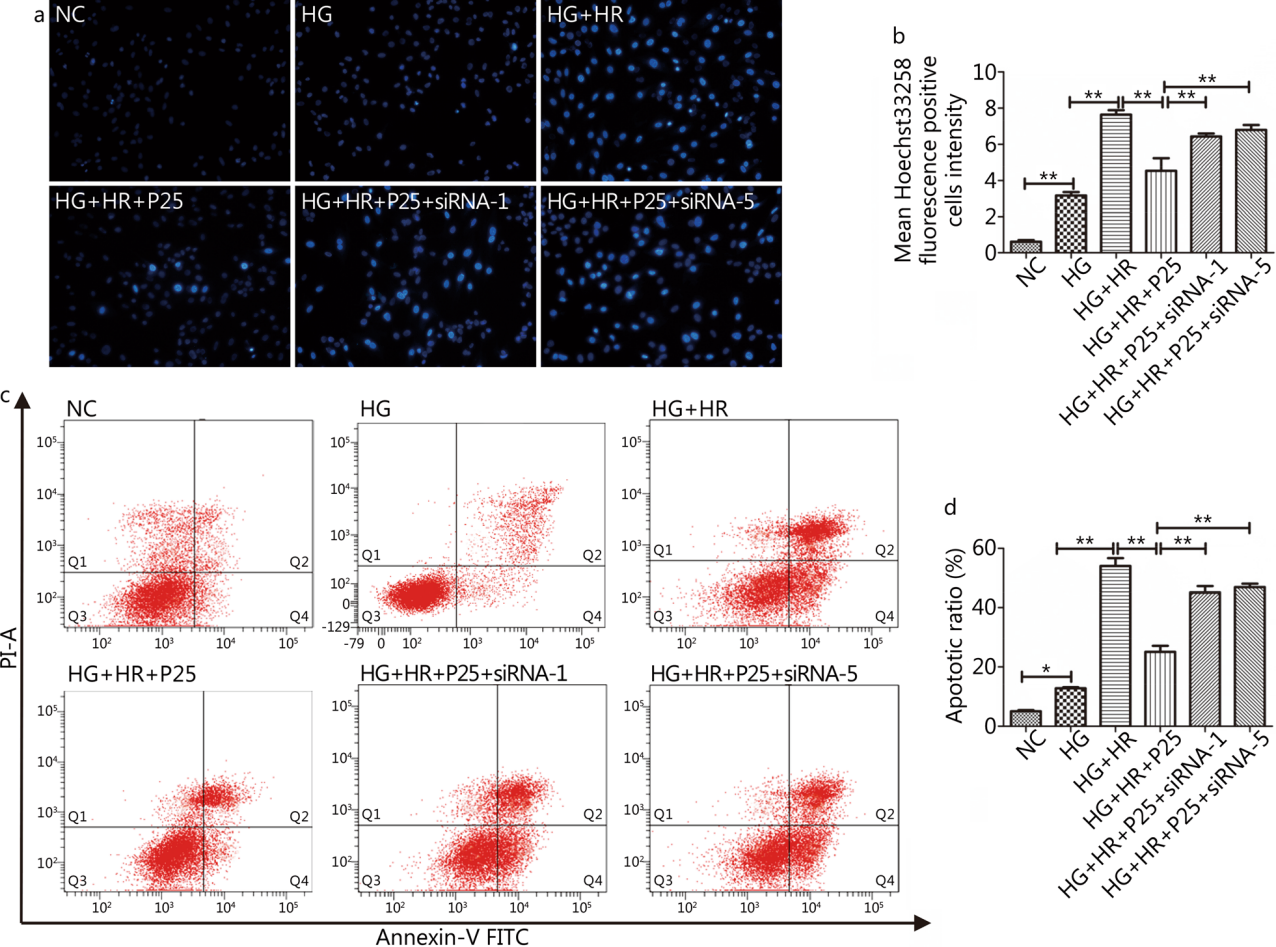
Inhibition of apoptosis ratio by propofol at the concentration 25 µmol/L were canceled by silencing of FoxO1 or FoxO3a. **a** Apoptotic cells marked by Hoechst 33,258 staining(200X). **b** Counts of early and late apoptotic cells by Annexin-V/PI assay. **c** Mean Hoechst 33,258 fluorescence positive intensity. **d** Representative image of apoptotic ratio. Data are mean ± SEM from three independent experiments each performed in triplicate. ^*^P < 0.05, ^**^P < 0.01

As shown in Figs. 7 and 8, P-PostC mediated reductions of ROS and apoptosis in H9c2 cells subjected to H/R under hyperglycemia were all canceled by silencing of FoxO1 (siRNA-1) or FoxO3a (siRNA-5). In Fig. 7, the mean fluorescence intensity of ROS in HG + HR + P25 + siRNA-1 group and HG + HR + P25 + siRNA-5 group were profoundly increased to the same level of HG + HR group (*P* > 0.05) but were significantly higher than those in the HG + HR + P25 group (all *P* < 0.05). Similarly, the degree of apoptotic cell death in the HG + HR + P25 + siRNA-1 group and HG + HR + P25 + siRNA-5 group were profoundly increased as compared to that in the HG + HR + P25 group (all *P* < 0.05, Fig. 8).

### Disruption of FoxO1 or FoxO3a canceled the protective effects of P-PostC in attenuating H/R induced increases in apoptosis and autophagy in H9c2 cells exposed to high glucose

**Fig. 9:**
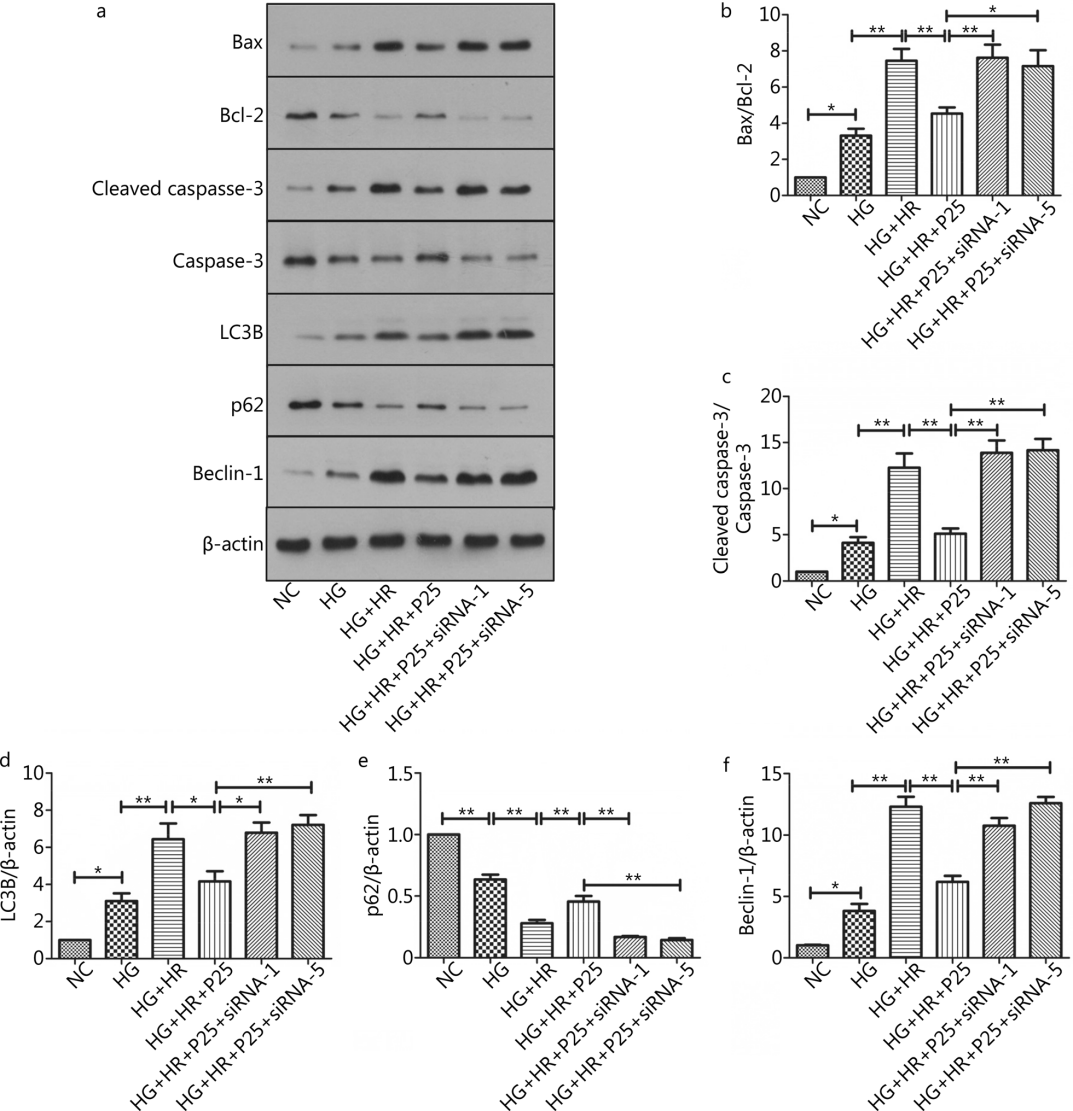
Disruption of FoxO1 or FoxO3a neutralized P-PostC (propofol-Postconditioning) protective effects against HR (hypoxia/reoxygenation) induced cells death under hyperglycemia. **a** Representative images of protein expression by western blotting. **b** Grey value of Bax/Bcl-2. **c** Ratio of cleaved caspase-3 to caspase-3. **d** Grey value of LC3B/β-actin. **e** p62 protein expression. **f** Beclin-1 protein expression. Data are mean ± SEM from three independent experiments each performed in triplicate. ^*^P < 0.05, ^**^P < 0.01

As depicted in Fig. 9, FoxO1 or FoxO3a gene knockdown canceled the protective effects of P-PostC in attenuating H/R induced increases in apoptosis and autophagy in H9c2 cells exposed to high glucose. When H9c2 cells were treated with HG, expression of Bax, cleaved caspase-3/caspase-3, LC3B and Beclin-1 were all significantly increased (all *P* < 0.05, HG *vs*. NC) while Bcl-2 and p62 significantly decreased and H/R further exacerbated these changes (all *P* < 0.05, HR *vs*. HG). All the above-mentioned disadvantageous changes induced by HG and HR were significantly attenuated by the administration of propofol at the early phase of reoxygenation (P-PostC) (all *P* < 0.05, HG + HR + P25 *vs*. HG + HR), but these beneficial effects of P-PostC were canceled by silencing of FoxO1 or FoxO3a (all *P* < 0.05, HG + HR + P25 *vs.* HG + HR + P25 + siRNA-1 or HG + HR + P25 + siRNA-5).

## Discussion

Consistent with the observed anti-apoptotic and oxidative stress effect of propofol in animal models reported previously [37–39], we showed that P-PostC significantly enhanced the survival of H9c2 cells in a dose-dependent manner and attenuated H/R induced increase of ROS in H9c2 cardiomyocytes exposed to hyperglycemia. The major advancement and novelty of the study is that the upregulation of the expressions of FoxO1 and FoxO3a play a key role in P-PostC mediated protection against H9c2 hypoxia/reoxygenation injury under the condition of high glucose. To our knowledge, our study is also the first to explore the roles of FoxO1 and FoxO3a simultaneously in vitro in cardiomyocytes in the context of hypoxia and reoxygenation and investigate their regulation on post-hypoxic cellular apoptosis and autophagy.

Evidence indicates that ROS are critically involved in the ischemia injury and it could be exaggerated during reperfusion by major oxidative stress systems such as the NADPH oxidase system and the xanthine oxidase system. The burst of ROS seen during reperfusion regulates a large number of vital pathways (survival/stress responses, apoptosis, autophagy, inflammatory response, necrosis, necroptosis, etc.) [40–43]. Diabetes or hyperglycemia induced increased production of ROS and the exacerbation of apoptosis during ischemia and reperfusion has been shown to be one of the major mechanisms that not only made the diabetic hearts more vulnerable to ischemic insult but also rendered the diabetic hearts less or not responsive to cardiac protective interventions that are otherwise effective in non-diabetic subjects [44–46]. Propofol has been discussed widely because of its inconsistent properties. It implied that propofol may attenuate I/R injury in vivo and in vitro due to its property as a ROS scavenger, and may also abolish pharmacologically-induced cardio protection [47]. The explicit reason for the above striking paradox is still unidentified, we assumed those discrepancies largely depend on the way of propofol administration (e.g., the timing and dosage of propofol application). Even though low to moderate level of ROS is considered as a self-defense mechanism, in our study, we found that cardiomyocytes H/R under hyperglycemia produced significantly oxidative stress accompanied with significant cell death, and, under such circumstance, P-PostC neutralized ROS induced cell injuries to confer protective effects.

Excessive apoptosis has long been regarded as a facilitator for the pathological progression of I/R myocardial injuries and inhibition of apoptosis thereby becomes a worthwhile approach to attenuate myocardial ischemia reperfusion injury [48–50]. It is well accepted that upregulation of cardiac specific Bcl-2 or downregulation of caspase-3 can significantly relieve I/R-induced cardiomyocytes apoptosis and infarct size [51, 52]. Likewise, autophagy also plays a dual role in the regulation of cardiomyocytes I/R injury [53–55]. It can promote cell survival by eliminating impaired cells but also provoke cell death through excessive degradation of essential cellular components with or without interaction of apoptosis [56–58]. Several published studies have suggested that autophagy is upregulated in the damaged heart [59, 60]. In the current research, high glucose increased the expressions of autophagy/apoptosis-related proteins, ranging from Bcl-2, Bax, capase-3, cleaved caspase-3, LC3B, p62 and Beclin-1, as well as being amplified by H/R injury. However, such H/R-induced increases of autophagy/apoptosis-related protein expressions were mitigated by P-PostC, and this should be the major mechanisms by which propofol increased the survival of H9c2 cells subjected to H/R under hyperglycemia.

ROS-mediated oxidative stress participates in a variety of signal transduction processes during myocardial I/R injury. FoxO family is not only the main meeting point of signaling including oxidative stress signaling but also the monitor of cell death pathways. Accumulated evidence has shown that the absence of FoxO1 can lead to incomplete development of blood vessels and results in embryonic cell death, while the knockout of FoxO3a can lead to inflammation of extensive organs and the disruption of lymphocyte proliferation [61–63]. Our experiments showed that 25 μmol/L of propofol post-treatment reduced the rate of apoptosis and autophagy caused by H/R injury in H9c2 cardiomyocytes exposed to high glucose, along with increased expressions of FoxO1 and FoxO3a. The above protective effect was reversed when either FoxO1 or FoxO3a was silenced. It is possible that propofol reduced the levels of ROS to a degree that can activate the FoxO family to subsequently confer antioxidant effects similarly as seen in neuron cells where stimulated low levels of oxidative stress activated the transcription factors FoxO1a and FoxO3a in the hippocampus of rats to confer cellular protection [64]. Our finding is in agreement with a previous study which showed that propofol could upregulate FoxO1 to attenuate myocardial cell injury after oxygen glucose deprivation and reperfusion (OGD/R) [28]. However, it is worth noting that in the study of Li et al., the authors showed that propofol produced cellular protection by inducing moderate autophagy in a H/R model in cardiomyocytes cultured under normal glucose and they used 6 h duration of hypoxia followed by 4 h of reoxygenation [18], which is different from our model and condition. It is generally known that moderate autophagy is a self-defense mechanism while excessive autophagy is detrimental. Under our experimental condition, autophagy was over activated, and thus inhibition of autophagy by propofol postconditioning conferred protective effects. Furthermore, studies in mammalian cells have revealed that a vital role of FoxO is to counteract oxidative stress, which is probably a character why FoxO can confer its prosurvival and longevity-promoting actions [30, 65]. Perhaps the specific activity of FoxO is primarily regulated by a complex and elaborated array of post‐translational modifications. Consequently, we found out that ROS-induced cellular injury, apoptosis, and autophagy under hyperglycemia were further exacerbated by silencing FoxO1 or FoxO3a, and that P-PostC could not confer cardiac protection in the absence of FoxO1 or FoxO3a. Findings of our in vitro studies provide a clue that activation of FoxO1 and/or FoxO3a may prove to be an effective approach in combating myocardial ischemia reperfusion injury in diabetes, despite that further studies in in vivo models of myocardial I/R is merited.

Patients outside the hospital attacked with acute myocardial ischemia generally needed more time before receiving specialized medical treatment and cardiac protective procedures like ischemic preconditioning is not applicable under this circumstance. Our finding that application of a high dose of propofol at the beginning of post-hypoxic reoxygenation could attenuate cardiomyocytes H/R injury even under high glucose may prove to be clinically applicable in situations after thrombolytic therapy, percutaneous coronary intervention (PCI) and coronary artery bypass surgeries when reperfusion injury is severe and glucose level is increased under stress. However, it should be noted that all our experimental data were confined to the degree of cells in vitro, and the investigation between propofol and FoxO1 or FoxO3a was limited to the regulation of the protein level. As a result, more in-depth research about FoxO family mediated cell death pathways and P-PostC still deserves future verification in animal diabetes models and clinical practices.

## Conclusions

Based on the findings, propofol post-treatment promotes cell survival through inhibiting apoptosis and autophagy induced by hypoxia/reoxygenation in H9c2 cells *under hyperglycemia,* and that activation of FoxO1 and FoxO3a expression is key for propofol to confer such cardiac protection. Propofol is widely utilized in various surgical procedures and intensive care units due to the speedy onset, rapid and complete recovery characteristics. As such, propofol is particularly suitable for wounded soldiers suffering from ischemia who need urgent treatments.

## Data Availability

Data collected and analyzed for the study are available from the corresponding author upon reasonable request.

## Abbreviations

CCK-8: Cell counting kit-8
CK-MB: Creatine kinase-MB
CST: Cell Signaling Technology
cTnI: Cardiac troponin I
DCFH-DA: 2, 7-Dichlorodihydrofluorescein diacetate
DHE: Dihydroethidium
DM: Diabetes mellitus
DMEM: Dulbecco's modified eagle medium
DMSO: Dimethyl sulfoxide
FoxO: Class O of forkhead box transcription factors
H/R: Hypoxia/reoxygenation
HG: High glucose
I/R: Ischemic reperfusion
LDH: Lactate dehydrogenase
MDA: Malondialdehyde
MnSOD: Manganese superoxide dismutase
OD: Optical density
PBS: Phosphate buffered saline
PCI: Percutaneous coronary intervention
P-PostC: Propofol postconditioning
RIPA: Radio immunoprecipitation assay
ROS: Reactive oxygen species
SEM: Standard error of mean
T-SOD: Total superoxide dismutase
